# Supplementary material for: Broadly neutralizing antibody epitopes on HIV-1 particles are exposed after virus interaction with host cells
Source: J Virol. 2023 Sep 28;97(9):e00710-23. doi: 10.1128/jvi.00710-23 (PMC10537810; doi:10.1128/jvi.00710-23)
Supplement: Supplemental figures — Figures S1 to S11. [file jvi.00710-23-s0001.pdf]

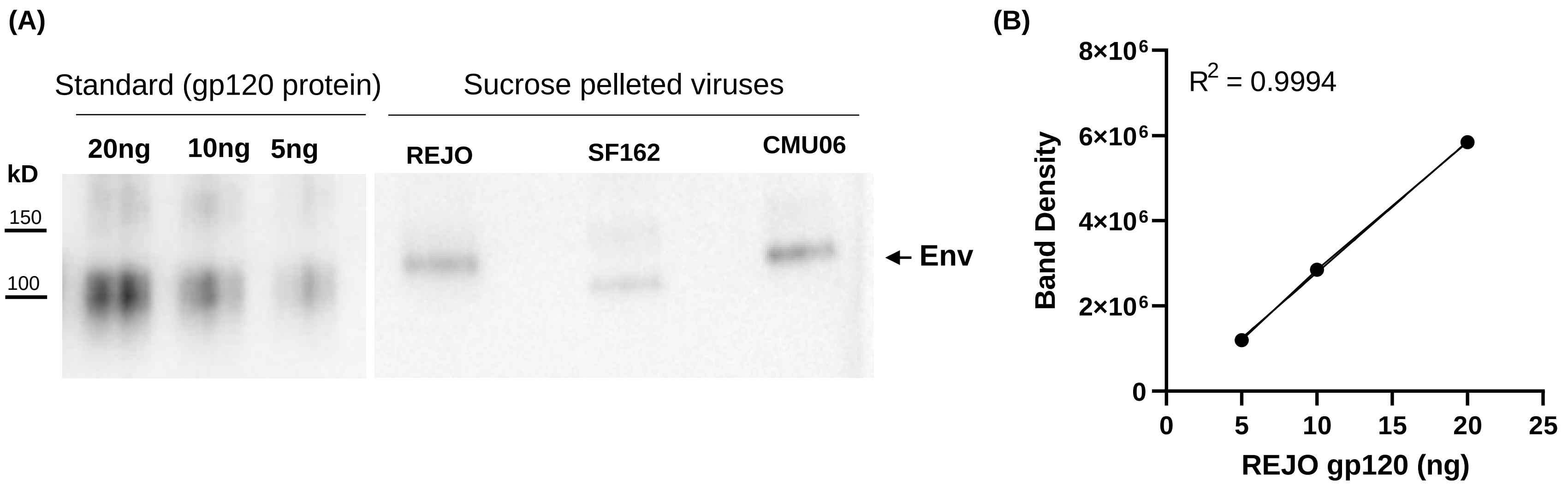

Supplementary Figure 1. Measurement of Env in virus preparations by Western blot. (A) Viruses produced in 293T cells, were concentrated (20X) by sucrose pelleting. Four µl of each 20X concentrated virus particle prep were lysed and analyzed by SDS-PAGE (4–20%) and Western blot. An anti-gp120 MAb cocktail (V3: 391/95-D, 694/98-D, 2219, 2558; C2: 847-D, 1006-30D; C5: 450-D, 670-D) was used to quantitate the levels of Env associated with virions. REJO gp120 protein loaded at different concentrations was used as a standard. The band density of the REJO gp120 protein was used to generate the linear curve (B) and to calculate the amount of Env in each virus preparation.

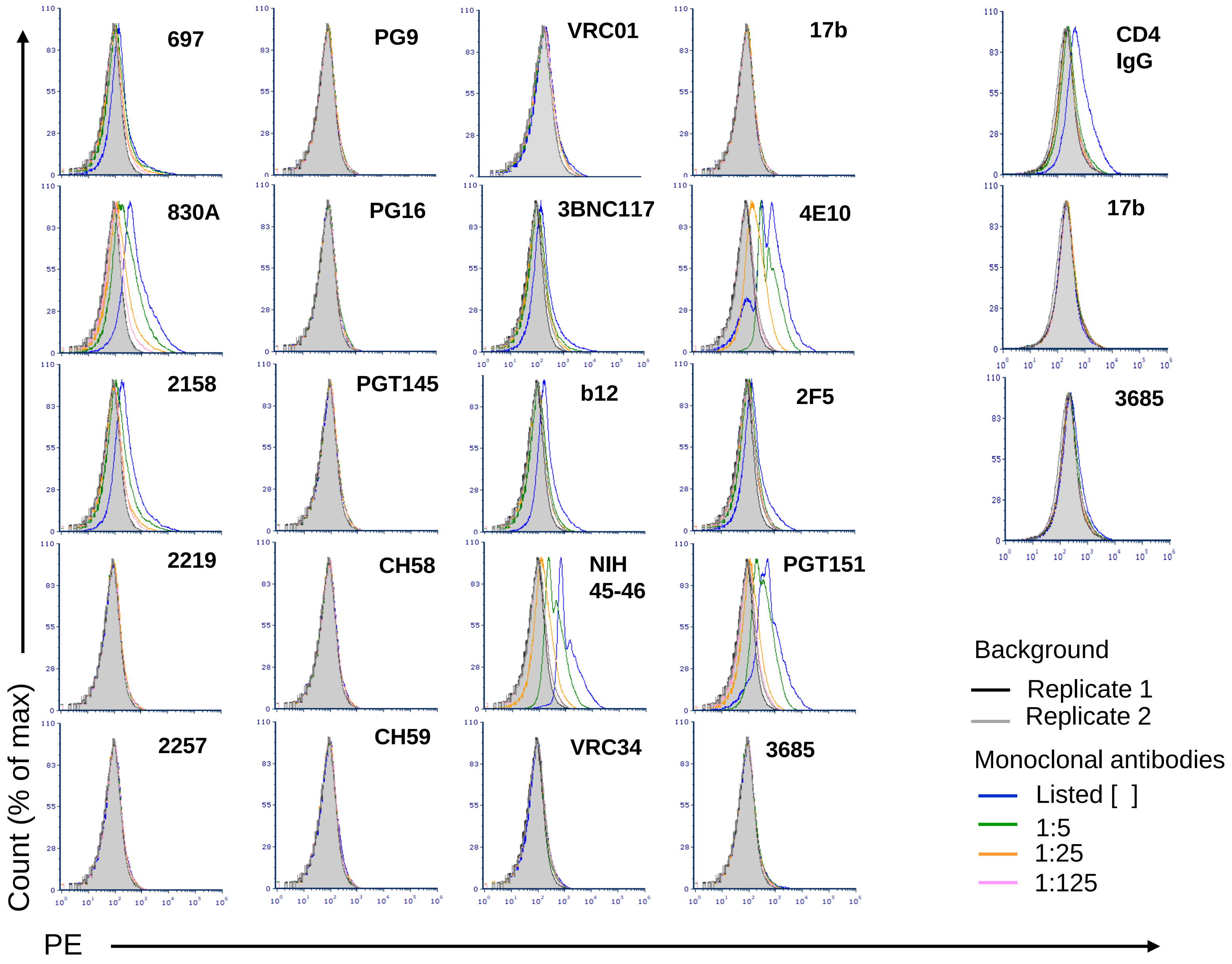

Supplementary Figure 2. Histogram plots related to Fig 2 showing reactivity of serially diluted mAbs to REJO virus particles coupled to fluorescent microspheres. Virus coupled microspheres stained with biotin and PE alone were used to set the background staining (gray). Experiment was repeated to probe for binding of CD4-IgG, 17b and negative control mAb 3685 (Right panel)

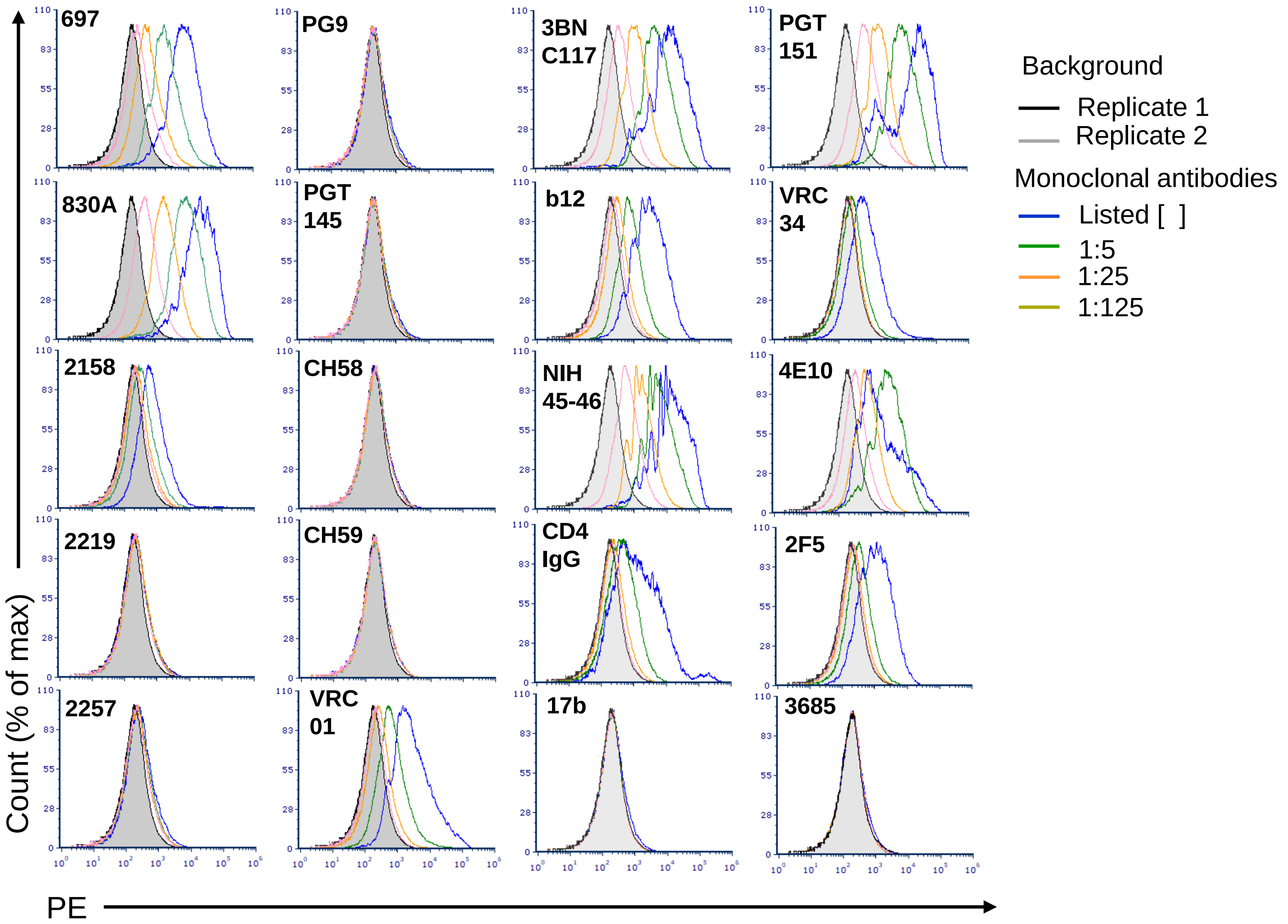

Supplementary Figure 3. Histogram plots related to Fig 3 showing reactivity of serially diluted mAbs to SF162 virus particles coupled to fluorescent microspheres. Virus coupled microspheres stained with biotin and PE alone were used to set the background staining (gray).

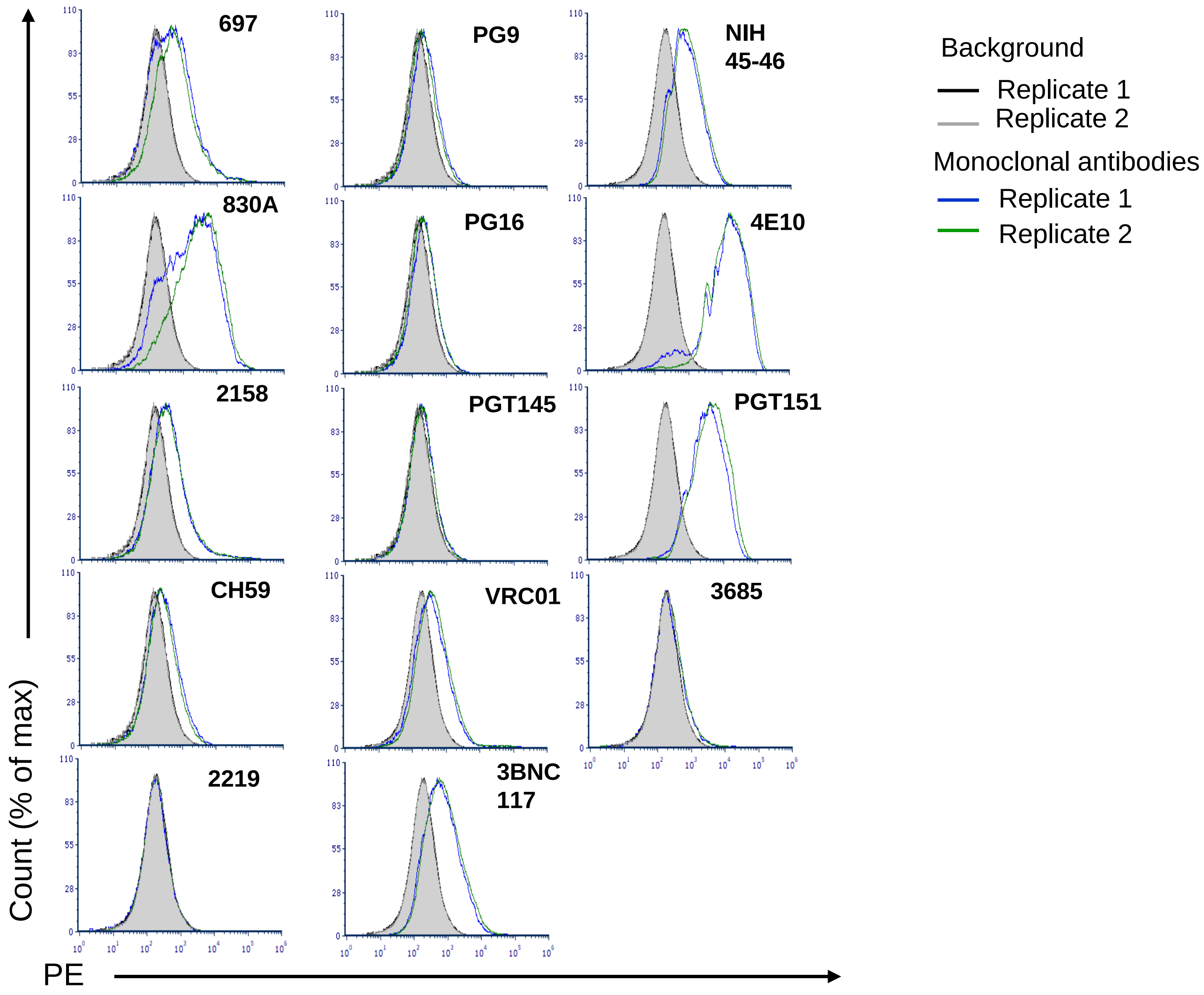

Supplementary Figure 4. Representative histogram plots related to Fig 4 showing reactivity of mAbs to CMU06 virus particles coupled to fluorescent microspheres. Binding was detected with streptavidin-PE and fluorescent intensity (MFI) are shown. Virus coupled microspheres stained with biotin and PE alone were used to set the background staining (gray).

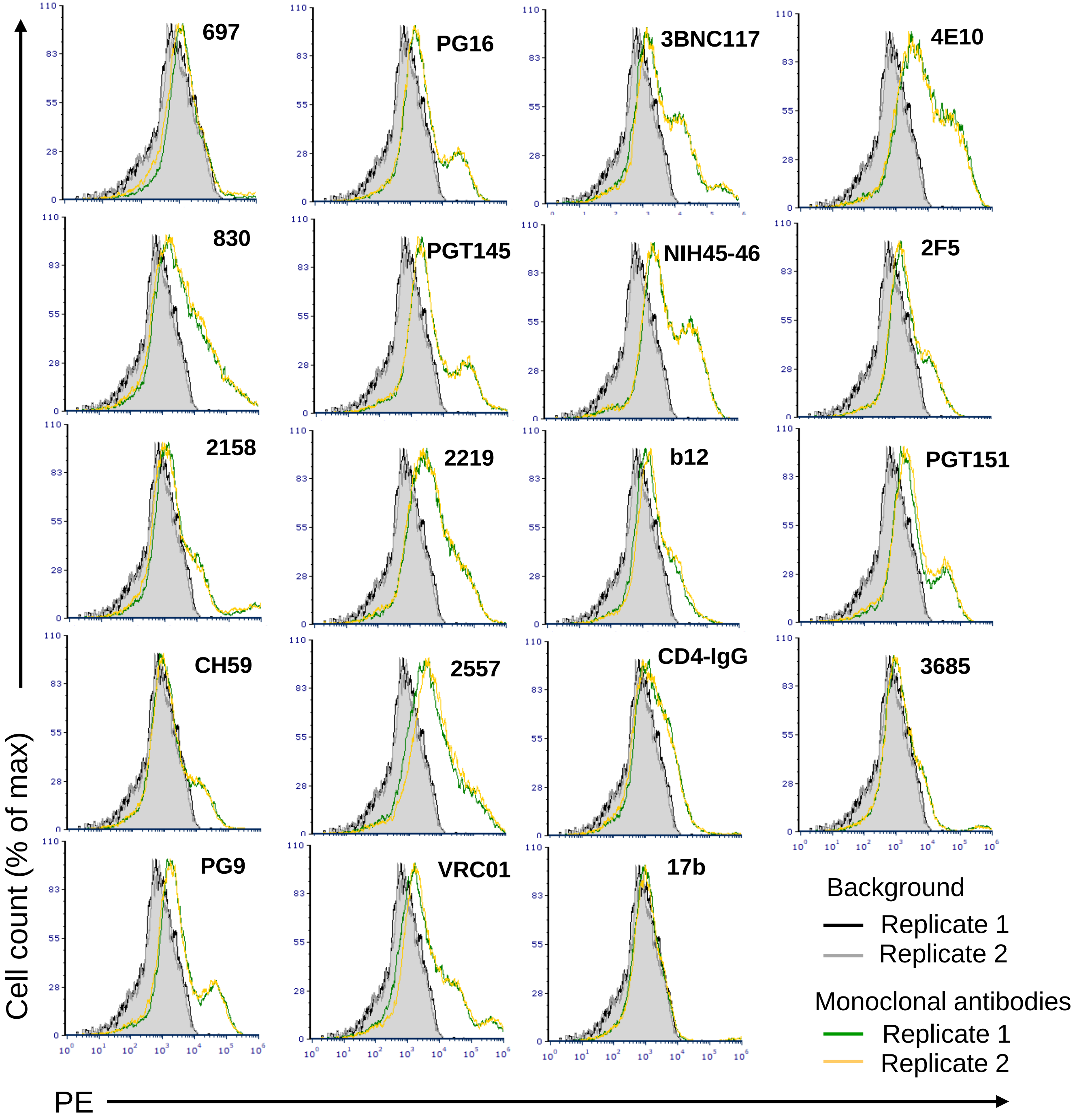

Supplementary Figure 5. Histogram plots related to Fig 5A showing reactivity of mAbs to REJO Env expressed on cell surface. Replicates from one representative experiment are shown. Binding was detected with streptavidin-PE. Transfected cells stained with biotin and PE alone were used to set the background MFI (gray). MAb 3685 was used as negative control.

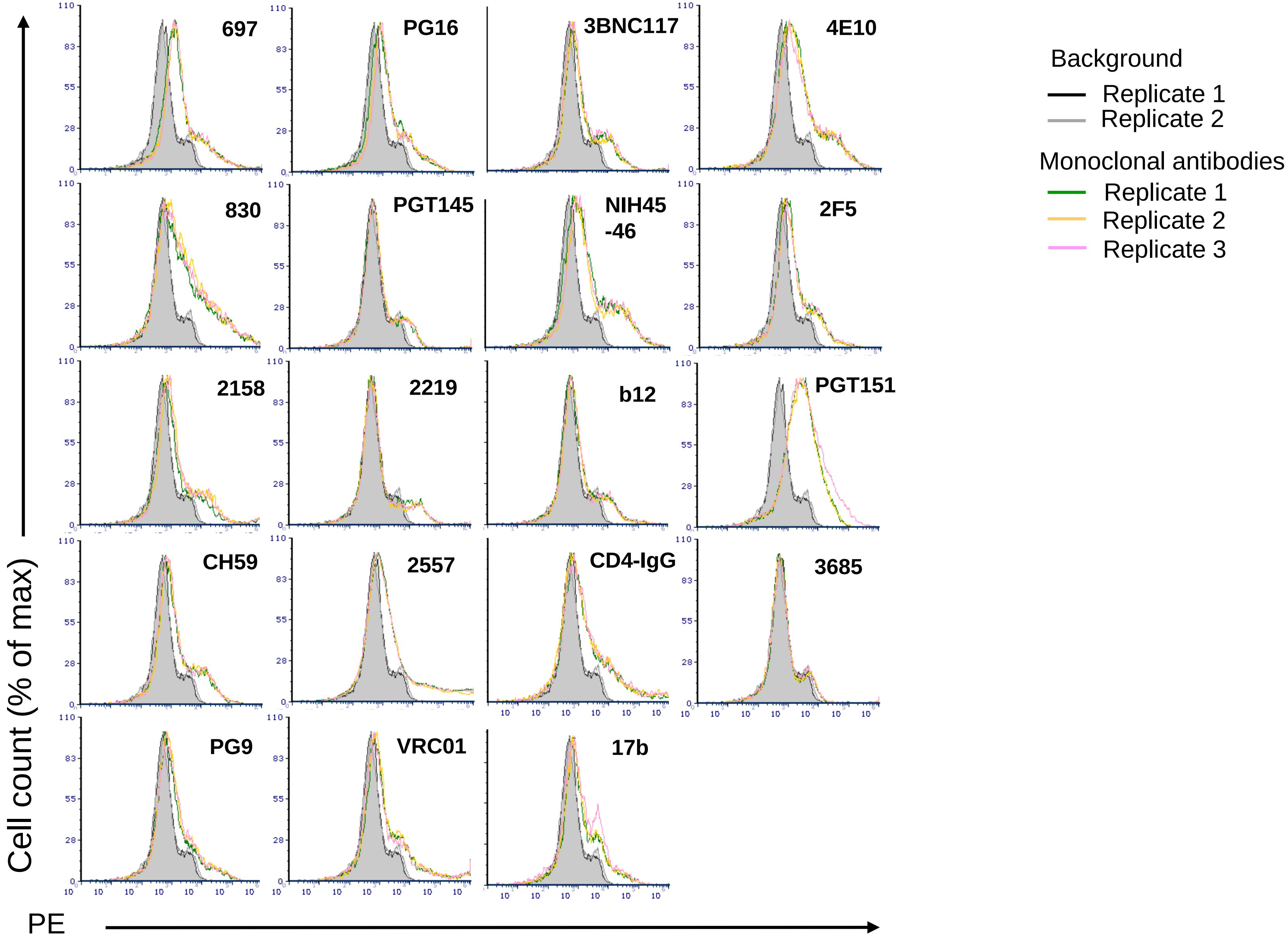

Supplementary Figure 6. Histogram plots related to Fig 5B showing reactivity of mAbs to CMU06 Env expressed on cell surface. Triplicates are shown. Binding was detected with streptavidin-PE. Transfected cells stained with biotin and PE alone were used to set the background MFI (gray). MAb 3685 was used as negative control.

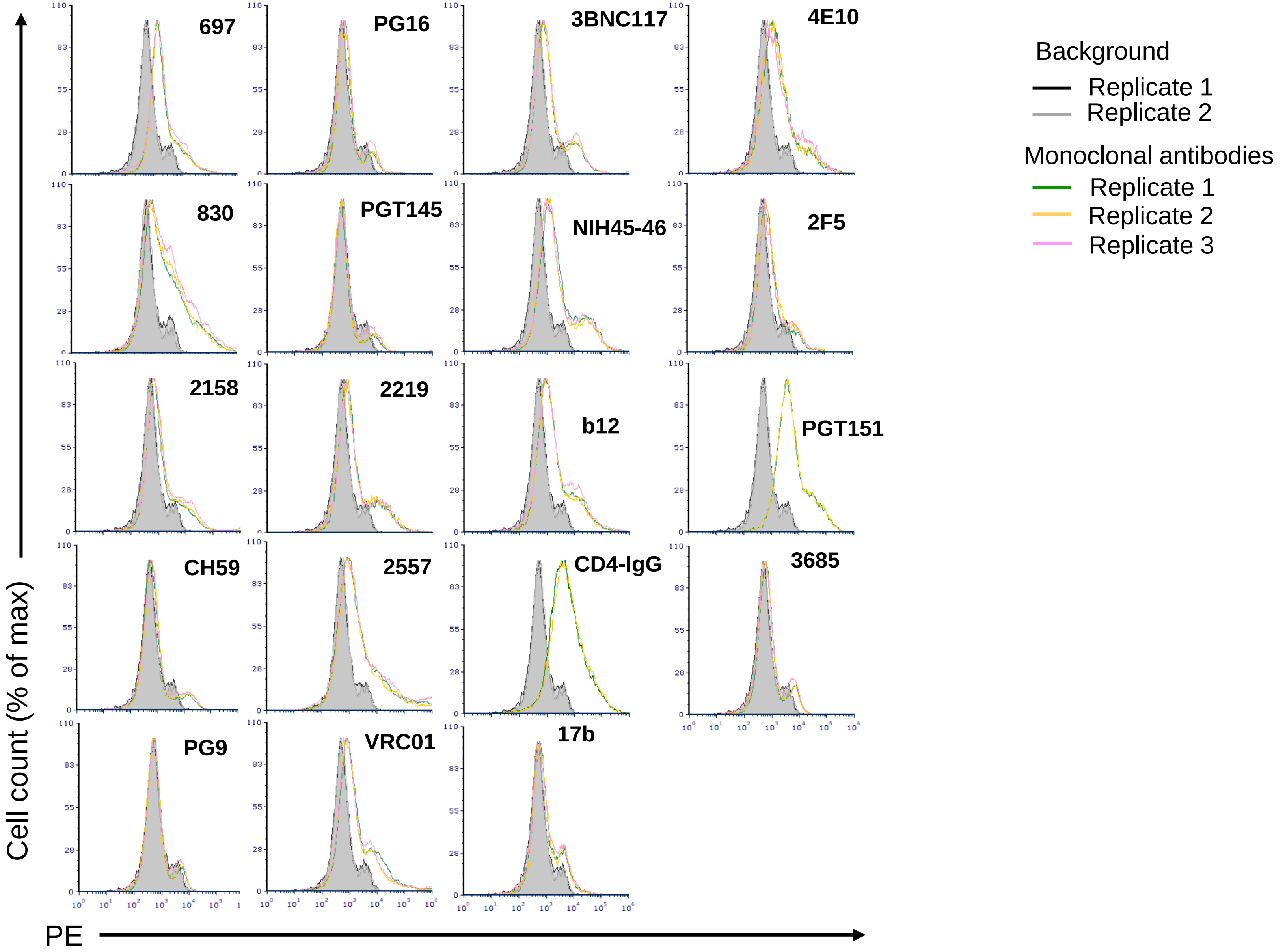

Supplementary Figure 7. Histogram plots related to Fig 5C showing reactivity of mAbs to SF162 Env expressed on cell surface. Triplicates for all antibodies and duplicates for CD4-IgG and PGT151 are shown. Binding was detected with streptavidin-PE. Transfected cells stained with biotin and PE alone were used to set the background MFI (gray). MAb 3685 was used as negative control.

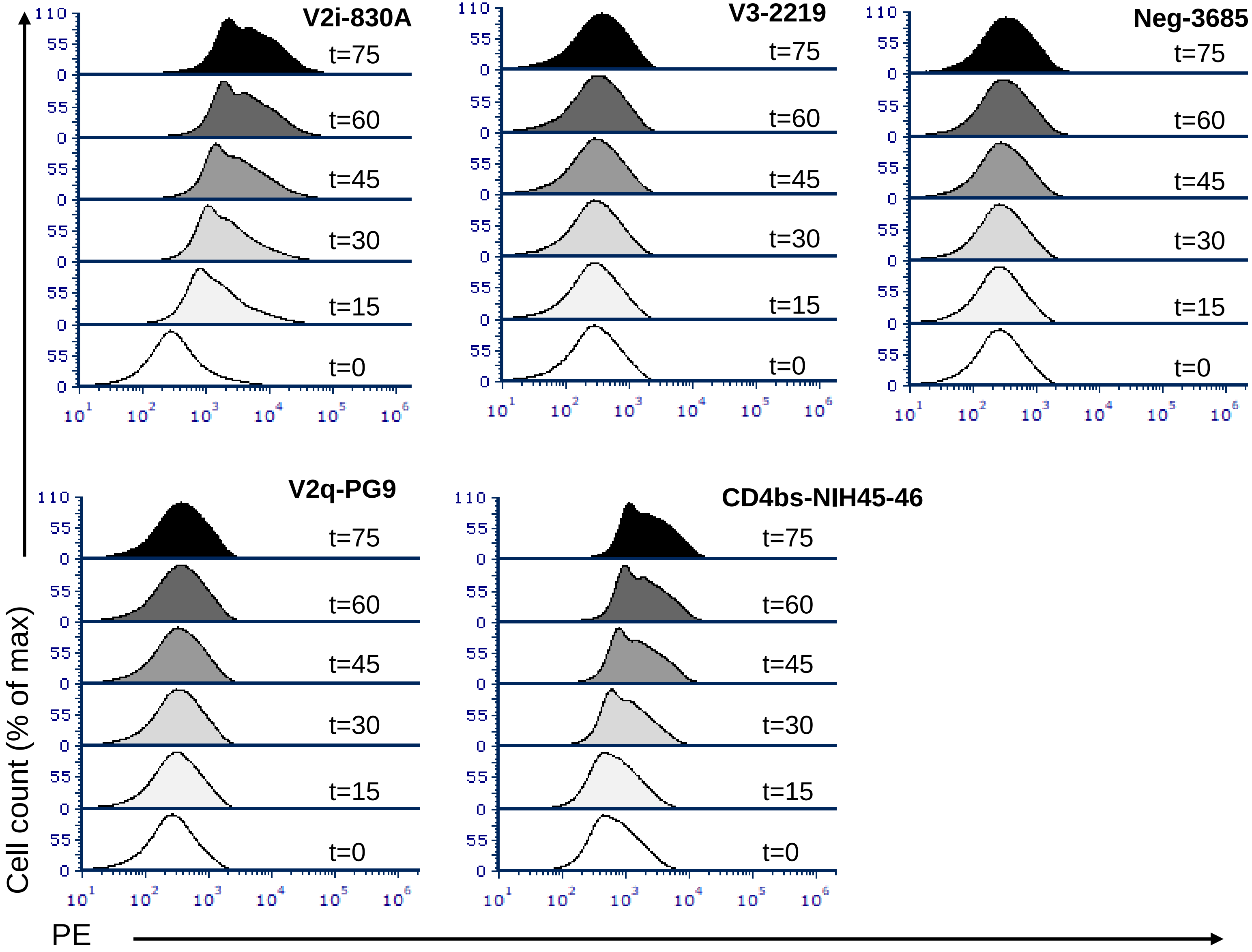

Supplementary Figure 8. Representative histogram plots related to Fig 6 showing time-dependent changes in reactivity of mAbs. Microspheres coupled to REJO virus particles were treated with mAbs at 37°C for various time from 0 to 75 min. Binding was detected with streptavidin-PE.

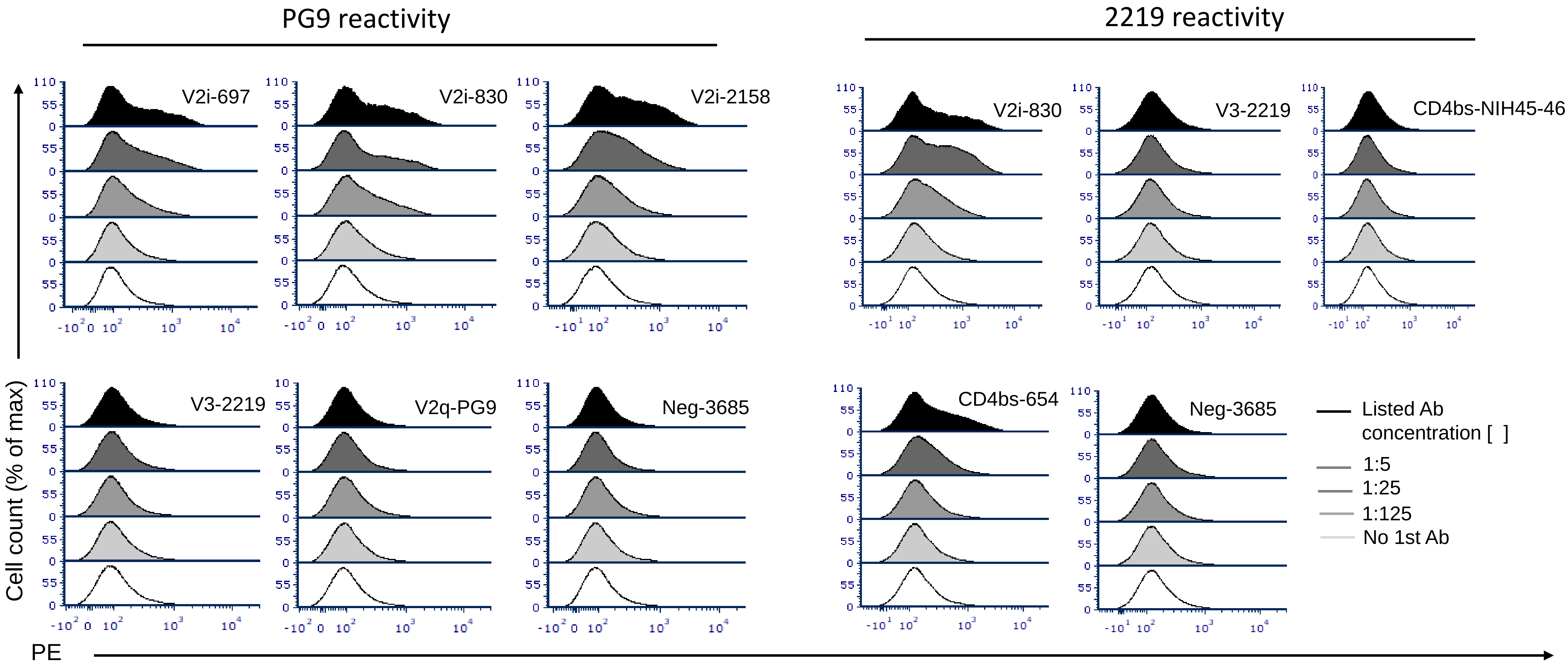

Supplementary Figure 9. Representative histogram plots related to Fig 7 showing changes in reactivity of PG9 and 2219 mAbs by different antibodies. Microspheres coupled with REJO virus were incubated with five-fold titrated antibodies followed by biotinylated V2q mAb PG9 (25 μg/ml). Binding was detected with streptavidin-PE.

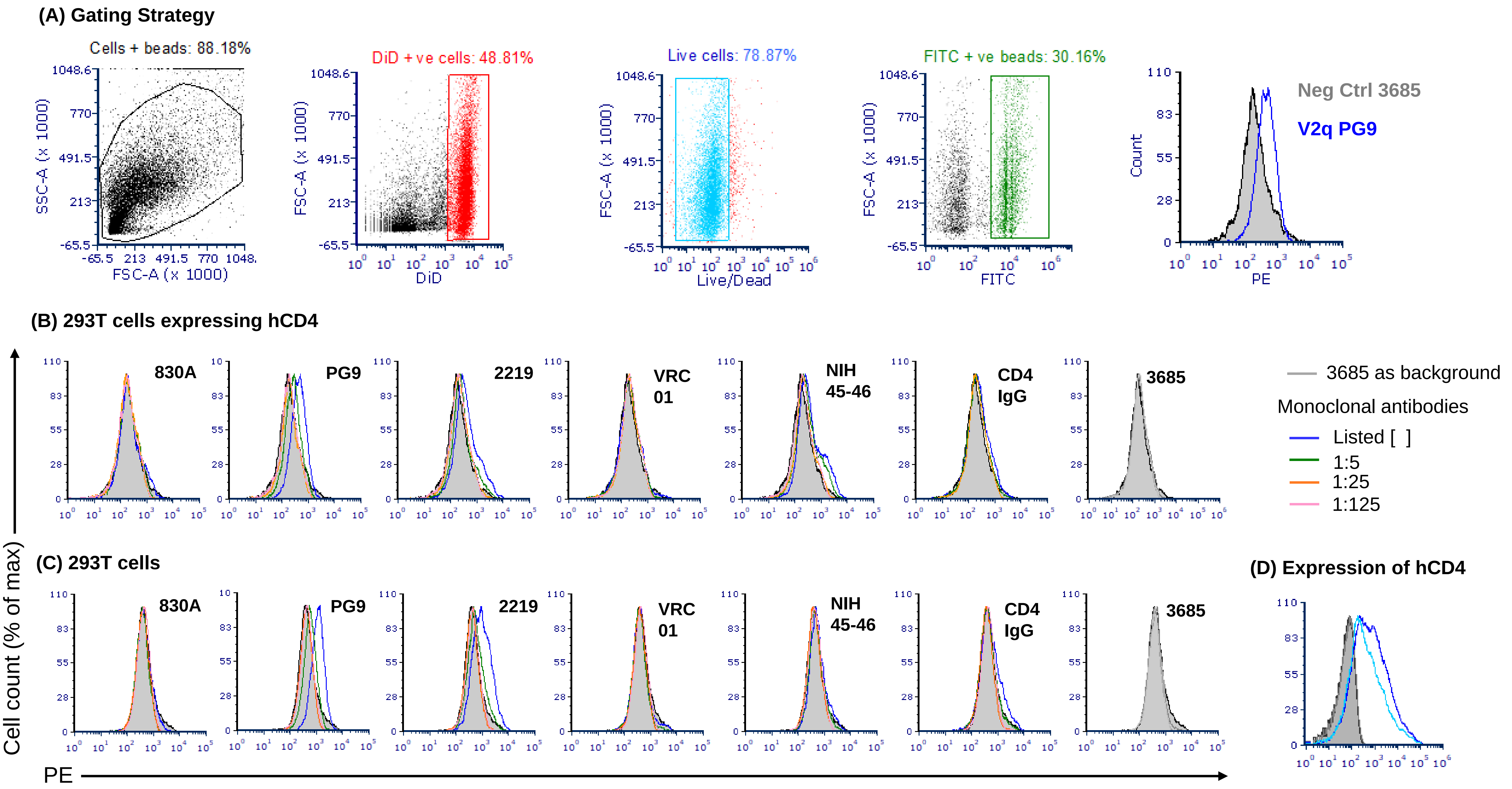

Supplementary Figure 10. Gating strategy (A) **and histograms related to Fig 10.** Plots showing binding of mAbs to virions in presence of (B) 293T cells transfected to express hCD4 and (C) un-transfected 293T cells. (D) Histograms showing cell surface expression of human CD4 293T cells. Cells expressing hCD4 are shown in blue and un-transfected cells are shown in gray.

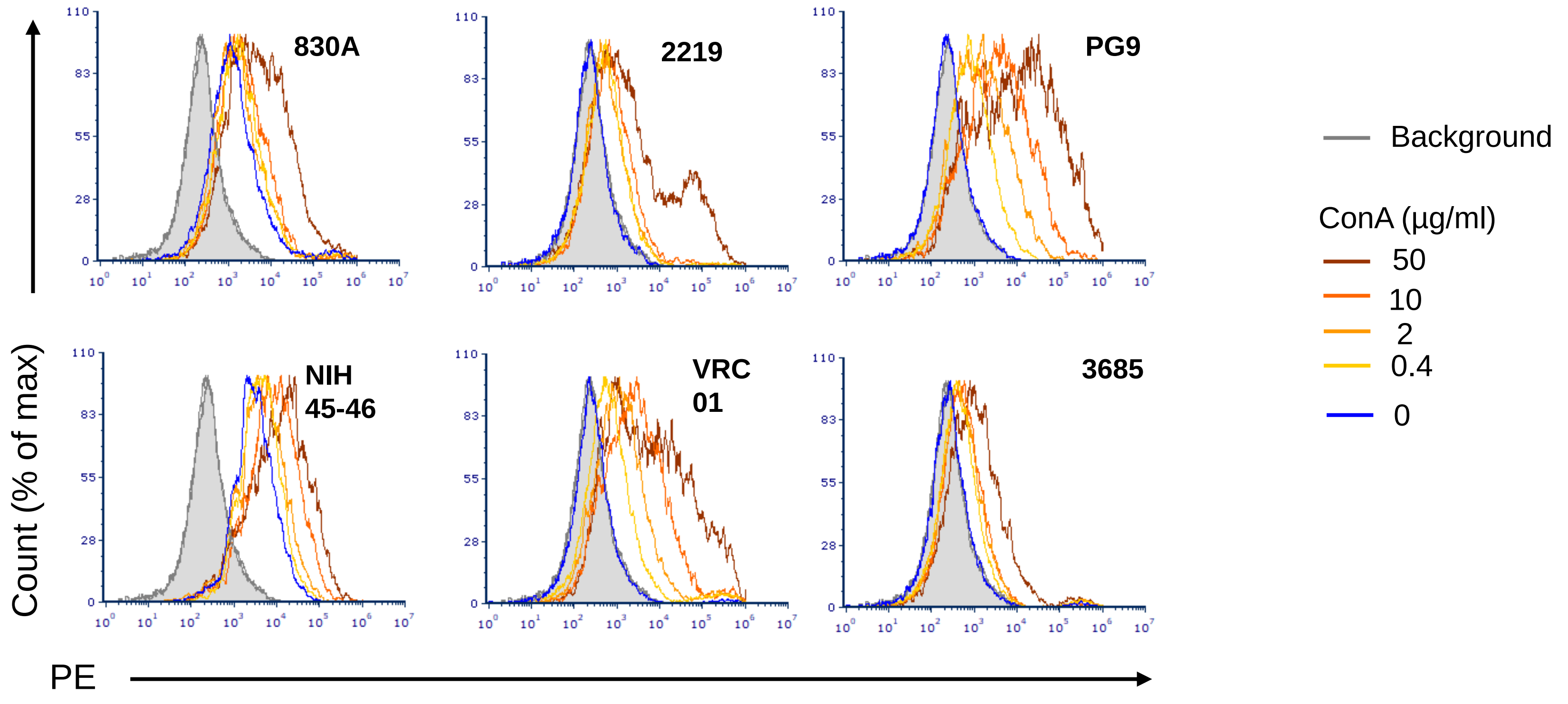

Supplementary Figure 11. Representative histogram plots related to Fig 11 showing changes in reactivity of mAbs by Concanavalin A (ConA).
